# Cigarette smoking: an important renal risk factor – far beyond carcinogenesis

**DOI:** 10.1186/1617-9625-1-11

**Published:** 2002-06-15

**Authors:** SR Orth

**Affiliations:** 1 Division of Nephrology and Hypertension, University Hospital Berne (Inselspital), Berne, Switzerland

## Abstract

In recent years, it has become apparent that smoking has a negative impact on renal function, being one of the most important remediable renal risk factors. It has been clearly shown that the risk for high-normal urinary albumin excretion and microalbuminuria is increased in smoking compared to non-smoking subjects of the general population. Data from the Multiple Risk Factor Intervention Trial (MRFIT) indicate that at least in males, smoking increases the risk to reach end-stage renal failure. Smoking is particularly "nephrotoxic" in older subjects, subjects with essential hypertension and patients with preexisting renal disease. Of interest, the magnitude of the adverse renal effect of smoking seems to be independent of the underlying renal disease. Death-censored renal graft survival is decreased in smokers, indicating that smoking also damages the renal transplant. Cessation of smoking has been show to reduce the rate of progression of renal failure both in patients with renal disease or a renal transplant. The mechanisms of smoking-induced renal damage are only partly understood and comprise acute hemodynamic (e.g., increase in blood pressure and presumably intraglomerular pressure) and chronic effects (e.g., endothelial cell dysfunction). Renal failure per se leads to an increased cardiovascular risk. The latter is further aggravated by smoking. Particularly survival of smokers with diabetes mellitus on hemodialysis is abysmal. In the present review article the current state of knowledge about the renal risks of smoking is reviewed. It is the aim of the article to point out that smoking not only increases the risk of renal cell carcinoma or uroepithelial cell carcinoma, but also the risk of a faster decline of renal function. The latter is a relatively new negative aspect which has not been widely recognized.

## Introduction

The fact that smoking is a risk factor for renal and uroepithelial cell carcinoma is well known. In the following, I shall review the current knowledge about the less well known adverse renal effects of smoking, which are of particular importance not only for nephrologists but physicians in general. Furthermore, I shall discuss the cardiovacular complications conferred by smoking in the renal patient.

Although earlier reports [1] had indicated that smoking may alter renal function, it was not until 1978 that additional information was published clearly indicating that smoking is a renal risk factor. At that time, Christiansen [2] documented that smoking confers an increased risk for the development of diabetic nephropathy in patients with type 1 diabetes. Thereafter, several studies, mostly performed by diabetologists, confirmed the finding of Christiansen even in patients with type 2 diabetes (for review see[3,4]). It is astonishing that it took 19 years until nephrologists became aware that smoking is a major renal risk factor. At that time the hypothesis was raised that smoking has a negative impact on renal function, independent of the pre-existing renal disease [5]. Since then, the interest into the topic and the knowledge about the adverse renal effects of smoking has continuously increased. Although large prospective studies are lacking, there is clear epidemiological evidence that smoking has to be considered as one of the most important renal risk factors. This is particularly true for patients with pre-existing renal disease and elderly hypertensive men, but recent evidence suggests that smoking may also impair renal function in patients with apparently normal kidneys. The epidemiological evidence for an adverse renal effect of smoking is summarised in Table 1 and will be discussed in detail (vide infra). Of importance in the context of patient management, there is good evidence that smoking cessation is probably the single most effective measure to retard progression of renal failure [6].

**Table 1:** Epidemiologic evidence for smoking-induced impairment of renal function

• Dose-dependent increase of urinary albumin excretion rate/proteinuria in cigarette smokers
• Dose-dependent increase of the risk of end-stage renal failure in male cigarette smokers of the general population
• Independent predictor of (micro)albuminuria in patients with primary hypertension
• Most powerful predictor of renal functional decline in patients with primary hypertension
• Increased risk of progression of renal failure in patients with primary renal disease
• In type 1 and type 2 diabetes mellitus: independent risk factor for the onset of microalbuminuria, for progression of microalbuminuria to manifest proteinuria
(i.e., diabetic nephropathy) and for acceleration of the rate of progression of diabetic nephropathy to end-stage renal failure
• Increased risk of renal allograft loss

Several potential mechanisms of smoking-induced renal damage have been discussed [7], e.g. increase in blood pressure, alteration of intrarenal hemodynamics, as well as activation of the sympathetic nerve, the renin-angiotensin and the endothelin systems. The pathomechanisms are, however, only partly understood. Recent evidence is available documenting that smoking induces structural alterations of the kidney in both humans and animal models.

Once in end-stage renal failure (ESRF), the failure to discontinue smoking adversely affects the prognosis of patients on renal replacement therapy and patients with a kidney transplant, mainly by increasing the risk of cardiovascular complications. Discontinuation of smoking has been shown to be the single most effective measure to prolong life [8]. This is particularly true in cardiovascular high risk patients such as patients with impaired kidney function.

## Effect of Smoking on Urinary Albumin/protein Excretion in Subjects of the General Population with Apparently Normal Kidney Function

The nephrotoxic effect of smoking in the general population is documented by a cross-sectional study in 7,476 non-diabetic subjects in Groningen, The Netherlands [9]. The study found that urinary albumin excretion rate correlates to the amount of cigarettes smoked per day. After adjustment for several potential confounding factors, subjects who smoked <20 cigarettes/day and subjects who smoked >20 cigarettes/day, respectively, showed a dose-dependent association between smoking and high normal albuminuria (relative risk: 1.33 and 1.98, respectively) and microalbuminuria (relative risk: 1.92 and 2.15, respectively). Interestingly, in a study including 40,619 subjects aged 28–75 years, smoking was associated even with increased urinary albumin concentrations far below the microalbuminuric range [10]. Analysis of a well defined non-diabetic and non-hypertensive subgroup of this latter study revealed that smoking was still independently associated with microalbuminuria [11]. Halimi et al. [12] documented a marked risk of irreversible proteinuria that may occur despite moderate smoking in a study including 28,409 subjects in France. These results from the Netherlands and France have recently been confirmed by a preliminary report documenting an association of smoking and albuminuria in a large cross-sectional probability sample of adults in the USA [13].

## Effect of Smoking on Renal Function in Subjects of the General Population with Apparently Normal Kidneys

The question arises whether the increase in albuminuria/proteinuria attributable to smoking is paralleled by an increased risk for renal functional deterioration.

This important issue was addressed in the study of Halimi et al. [12]. Smokers did not exhibit lower creatinine clearance than never smokers. Creatinine clearance was even slightly higher in current smokers, at least in men, even when normotensive and hypertensive subjects were analysed separately. The difference was, however, small, particularly in women. The effect of current smoking on creatinine clearance was reversible upon smoking discontinuation. This observation is compatible with smoking-induced early hyperfiltration. Data from the prospective Multiple Risk Factor Intervention Trial (MRFIT) including 332,544 men, indicate, however, that smoking also increases the risk of ESRF in the general male population [14]: a dose-dependent increase of the relative risk for ESRF was found in smokers as compared to non-smokers (up to 1.69 for heavy smokers) [15]. The increase in risk was independent of age, ethnicity, income, blood pressure, diabetes mellitus, prior history of myocardial infarction, or serum-cholesterol. Unfortuantely, the data concerning the relative risk of ESRF attributable to smoking have never been published as a full-size paper. Additional information is only available from a retrospective case-control study analysing data obtained from 4,142 non-diabetic participants of the Cardiovascular Health Study Cohort, all at least 65 years of age, who had two measurements of serum-creatinine performed at least three years apart [16]. In this elderly population the number of cigarettes smoked per day was highly associated with an increase in serum creatinine >27 μmol/L (>0.3 mg/dL). The definition for renal functional deterioration in this study is undoubtedly weak, but smoking may be one of the factors explaining why an impairment of renal function is observed in some, but not all elderly [17,18]. This assumption is in line with the observation in a sample of 455 adults in Wadena, Minnesota [19], where the decrease in creatinine clearance was greater in ex-smokers and current smokers than in non-smokers.

It can be concluded that (i) smoking increases the risk of albuminuria/proteinuria in the general population and (ii) that there is some evidence indicating that smoking increases the risk of renal functional impairment in the general population, particularly in men and in the elderly. The definition of renal functional deterioration in the studies available is, however, not beyond any doubt and large prospective studies investigating hard end-points, e.g. time to doubling of serum-creatinine, are clearly indicated.

It is noteworthy that the negative impact of smoking on renal function contributes to the increased cardiovascular risk conferred by smoking. An increase in urinary albumin concentrations far below the microalbuminuric range is associated with increased prevalence of established cardiovaskular risk factors and cardiovascular morbidity in the general population [10]. This is true even in non-diabetic and non-hypertensive subjects [11].

## Adverse Renal Effects of Smoking in Patients with Primary Hypertension

Proteinuria is found in 4–18% and albuminuria in 10–25% of patients with primary hypertension [20,21]. Albuminuria (and even more proteinuria) is an independent predictor of cardiovascular mortality in patients with primary hypertension [21-26].

Smoking emerged as an independent predictor of (micro)albuminuria in several studies which examined otherwise healthy hypertensive subjects. Mimran et al. [27] studied lean patients with primary hypertension and found that the prevalence of microalbuminuria was almost double in smokers compared to non-smokers. Hörner et al. [28] confirmed this finding and found that smoking was the strongest predictor for albuminuria in patients with primary hypertension. The Heart Outcomes Prevention Evaluation (HOPE)-Study [29] documented that smoking was an independent determinant of microalbuminuria in all participants, i.e. non-diabetic and diabetic patients with a high cardiovascular risk profile (approximately 50% being hypertensive). Furthermore, a recent study [30] found that patients with hypertension and left ventricular hypertrophy smoking >20 cigarettes/d had a 1.6-fold higher prevalence of microalbuminuria and a 3.7-fold higher prevalence of macroalbuminuria than never-smokers.

Important new information has become available concerning the negative impact of smoking on renal functional deterioration in hypertensive patients. Regalado et al. [31] performed a prospective study including 51 patients with primary hypertension for a mean follow-up of 35.5 months. Despite reduction of mean arterial blood pressure from 126.8 ± 1.3 mmHg to 96.5 ± 1.1 mmHg, plasma-creatinine increased from 133 ± 9 μmol/L (1.5 ± 0.1 mg/dL) to 168 ± 18 μmol/L (1.9 ± 0.2 mg/dL). Factors that independently predicted renal functional decline were smoking, greater initial plasma creatinine level, and black ethnicity. Smoking was by far the most powerful predictor of renal functional deterioration and the only one which is remediable. It is noteworthy that the mean increase in plasma-creatinine for the given observation period is more than can be expected in a representative sample of patients with primary hypertension, so that the data of this well performed, but small prospective study may not be generalisable. Indeed, a large prospective study [32] including 5,730 black and 6,182 non-black hypertensive male subjects (mean age 52.5 ± 10.2 years) from the Veterans Administration Hypertension Screening and Treatment Program clinics did not find a relation between smoking and the risk of ESRF during a minimum of 13.9 years of follow-up.

Thus, the issue whether or not smoking increases the rate of progression in patients with primary hypertension remains controversial. Considering the proven effects of smoking on albuminuria/proteinuria it is, however, cautious to conclude that smoking has to be considered as a renal risk factor in hypertensive patients.

## Adverse Renal Effects of Smoking in Patients with Renal Disease

The effect of smoking in patients with renal disease is of major interest, because it can be anticipated that this population is particularly susceptible to smoking-induced renal damage.

### Diabetic nephropathy

The first well documented reports about an increased renal risk in smokers were mostly retrospective studies in patients with type 1 diabetes. It was noted that smokers have a higher risk to develop diabetic nephropathy than non-smokers [2,33].

In the study of Telmer et al. [33] 668 patients with type 1 diabetes were investigated. The prevalence of diabetic nephropathy was significantly higher among "heavy" smokers (>10 cigarettes/d for more than one year) than among "non-heavy" smokers, i.e. 19.2% versus 12.1%. An increasing frequency of nephropathy was found with increasing cigarette consumption. Diabetic nephropathy was present in 13% of patients who smoked <10 cigarettes/d, but in >25% in patients who smoked 30 cigarettes/d. In a cross-sectional study Nórden and Nyberg [34] analysed smoking habits in 47 matched pairs of patients with type 1 diabetes with and without nephropathy. Patients with nephropathy had a significantly higher smoking index (number of cigarettes smoked/d multiplied by number of years smoking) than control patients. There were also significantly more current smokers (n = 22 versus n = 14), more heavy smokers (n = 20 versus n = 14), and fewer individuals who had never smoked (n = 9 versus n = 18) in the group with nephropathy compared to the group without nephropathy.

Since then, several studies confirmed an increased renal risk in patients with both type 1 and type 2 diabetes who smoke.

Smoking (i) increases the risk to develop microalbuminuria [29,35-45], (ii) accelerates the rate of progression from microalbuminuria to manifest proteinuria [46-51], and (iii) accelerates progression of renal failure [47,52-55].

An association between albuminuria/proteinuria and smoking has also been found among children with type 1 diabetes [40] and patients with type 1 diabetes who survived >30–40 years [56,57]. A genetic predisposition of smokers to develop albuminuria is suggested by the results of the BErgamo NEphrologic DIabetes Complications Trial (BENEDICT). The DD-genotype of the ACE gene was strongly associated with microalbuminuria in smokers [58].

As in non-diabetics, microalbuminuria and overt nephropathy are independent predictors of cardiovascular morbidity and mortality [59], adding to the increased cardiovascular risk conferred by smoking itself.

The magnitude of the effect of smoking on the risk to develop microalbuminuria in patients with diabetes mellitus is not minor. Chase et al. [35] reported that in a group of 359 young subjects with type 1 diabetes the prevalence of borderline (>7.6 μg/min) and abnormal (>30 μg/min) urinary albumin excretion rate was 2.8-fold higher in smokers than non-smokers. Similarly, the risk to have microalbuminuria short-term after the diagnosis of type 2 diabetes is highly increased in current smokers. The odds ratio for the presence of microalbuminuria was 26.3 for current smoking and 3.42 for a 1% increment in glycosylated HbA1 [44]. Although the confidence intervalls of these latter results were wide, the data indicate the importance of smoking compared to glycemic control as a classic renal risk factor in diabetes mellitus.

Concerning the risk to progress from microalbuminuria to gross proteinuria (>300 mg/d), a prospective study with an observation time of 4 years including 794 patients with type 2 diabetes reported a 2- to 2.5-fold higher relative risk in heavy smokers than in never-smokers [50].

The acceleration of the rate of progression of renal failure induced by smoking is dramatic. Sawicki et al. [53] calculated the adjusted odds ratio for progression of nephropathy in patients with type 1 diabetes. Progression was defined as an increase in proteinuria >20% and/or a reduction of glomerular filtration rate >20% after one year of follow-up. The odds ratio was 2.74 for each 10 cigarette pack years. In this study all patients were on intensified insulin and antihypertensive therapy, so that confounding effects of hyperglycemia and hypertension are minimised. In a study of Biesenbach et al. [52] the rate of loss of glomerular filtration rate was higher by a factor of 1.44 and 1.66 in smoking as compared to non-smoking patients with type 1 and type 2 diabetes, respectively. Thus, the influence of smoking on the rate of progression is similar in type 1 and type 2 diabetes.

A recent prospective study with a mean follow-up time of 5.3 years including 33 patients with type 2 diabetes and manifest nephropathy investigated the impact of smoking on progression of renal failure [60]. The initial serum-creatinine was 93 ± 7 μmol/L (1.05 ± 0.08 mg/dL) in smokers (n = 13) and 95 ± 3 μmol/L (1.08 ± 0.03 mg/dL) in non-smokers (n = 20). At the end of observation time, the increase of serum-creatinine was more pronounced in smokers as compared to non-smokers, i.e. 157 ± 18 μmol/L (1.78 ± 0.2 mg/dL) versus 117 ± 4 μmol/L (1.32 ± 0.04 mg/dL). This difference was not explained by potential confounding factors and regression anlaysis revealed that smoking was the only parameter that significantly predicted renal functional decline. These data are of particular importance, because blood pressure had been treated according to current standards including an ACE-inhibitor and achieving mean arterial blood pressure of 92 ± 1 mmHg. Thus, smoking seems to remain a renal risk factor despite lowering of blood pressure to the target level using currently recommended therapy, at least in patients with type 2 diabetes. A confirmation of the intreguing finding of a loss of the nephroprotective effect of ACE inhibitor-treatment in patients with type 2 diabetes who smoke is needed. Although not comparable, a retrospective case-control study investigating patients with primary renal disease [61] had reported that ACE inhibitor treatment counteracts the deleterious effect of smoking on renal function. An explanation why ACE inhibition may protect against smoking-induced renal functional decline may be the improvement of vascular dysfunction in smokers [62]. *In vitro *data suggest that this effect is partly mediated by scavenging free radicals and by attenuation of the cigarette-induced suppression of nitric oxide production [63]. Due to these considerations and the fact that despite low serum-renin, there is good evidence that the intrarenal angiotensin II production in patients with type 2 diabetes mellitus is increased [64], the above finding of Chuahirun et al. is contraintuitive [60].

If the data of the well performed but small study by Chuahiran et al. can be confirmed, it can be expected that smoking also counteracts the nephroprotective effect of blood pressure lowering including an ACE-inhibitor in patients with type 1 diabetes. This assumption is based on the fact that the course of diabetic nephropathy is identical in type 1 and type 2 diabetes [65] and that the adverse renal effect of smoking on renal functional decline are comparable in type 1 and type 2 diabetes [52].

It is of major clinical importance that smokers are at greater risk to develop type 2 diabetes [66-68]. In a prospective cohort study on 1266 non-diabetic males aged 35–59 years Nakanishi et al. [68] found that the relative risk to develop impaired fasting glucose during 5 years of observation was 1.62-fold higher in ever-smokers as compared to never-smokers. The relative risk to develop type 2 diabetes was dose-dependent: 1–20 cigarettes/d = 1.88 (CI: 0.71–5.0), 21–30 cigarettes/d = 3.02 (CI: 1.15–7.94), >31 cigarettes/d = 4.09 (CI: 1.62–10.29). The increased risk may be related to the fact that smoking aggravates insulin resistance in healthy smokers, at least according to some studies [7].

An interesting issue is the effect of smoking on the risk to develop proliferative retinopathy. Based on a priori considerations, one would expect that the risk is increased in smokers, since damage to the microvascular bed of the kidney, i.e. diabetic nephropathy, is often associated with damage to the microvascular bed of the eye, i.e. proliferative diabetic retinopathy [69-72]. In most [33,71,73-78] but not all studies [41,46,79,80], no difference in the prevalence of proliferative retinopathy was found between smokers and non-smokers with type 1 or type 2 diabetes. Vascular beds of the retina and the kidney have a different susceptibility for smoking-induced damage. The reasons for this difference are unknown. From the above studies it can be concluded that if smoking has any impact on the risk of onset and progression of diabetic retinopathy, this effect has to be considered as minor.

In summary, there is clear evidence that smoking has adverse effects on the onset and evolution of diabetic nephropathy in type 1 and type 2 diabetes mellitus. Furthermore, the number of cigarettes smoked per day and the number of pack-years of exposure seem to be associated with development of impaired fasting glucose and type 2 diabetes.

### Non-diabetic renal disease

There is no evidence in the literature that smoking induces any type of glomerulonephritis or any systemic disease involving the kidney to begin with [81-84]. Solid evidence has accumulated, however, that smoking is a major renal risk factor in patients with primary renal diseases.

In patients with autosomal dominant polycystic kidney disease (ADPKD) Chapman et al. [85] had found that individuals with established proteinuria had a significantly greater pack-year smoking history than did their nonproteinuric counterparts. Based on this first paper indicating an adverse renal effect of smoking in patients with primary renal disease, we performed a retrospective matched case-control study [61]. This European multicenter study was designed to assess whether smoking in patients with IgA-glomerulonephritis (IgA-GN) and ADPKD increases the risk to progress to ESRF. Because analysis of smoking (given as pack-years; PY) showed no strata inhomogeneity between renal diseases, IgA-GN and ADPKD were pooled for statistical analysis. Due to small sample size and modest average tobacco consumption, the subgroup of women was excluded from further analysis. Table 2 shows the distribution of cigarette smoking in male patients; consumption of cigarettes was subdivided into three categories, i.e., 0–5, 5–15 and >15 PY. The crude estimators for different quantitative levels of smoking document a dose-dependent increase in the risk for ESRF in male smokers as compared to non-smokers or moderate smokers (0–5 PY) (Table 2). After adjustment for possible confounders, multivariate analysis revealed that the risk for ESRF was substantially higher in male smokers with no history of ACE inhibitor treatment. In contrast, the odds ratio for ESRF of smokers with a history of ACE inhibitor treatment was not significantly increased (Table 3). Another case-control study confirmed that male patients with glomerulonephritis who smoke are at increased risk of renal function impairment [86].

**Table 2:** Crude smoking-associated risk of end-stagel renal failure in 144 male patients with IgA-glomerulonephritis or autosomal dominant poly- cystic kidney disease [61]

	Cases	Controls		95%-confidence intervall	
Pack-years	(n, [%])	(n, [%])	Odds ratio		p-value*
0–5	26 [36]	47 [65]	1.0	-	-
5–15	17 [24]	11 [15]	3.5	1.3–9.6	0.017
>15	29 [40]	14 [19]	5.8	2.0–17	0.001

* Wald χ^2^.

**Table 3:** Smoking-associated risk of end-stage renal failure (stratified for ACE inhibitor treatment and adjusted for systolic blood pressure) in 144 male patients with IgA-glomerulonephritis or autosomal dominant polycystic kidney disease [61]

Pack-years	Odds ratio	ACE inhibitor 95%-confidence intervall	p-value*	Odds ratio	No ACE inhibitor 95%-confidence intervall	p-value*
<5	1.0	-	-	1.0	-	-
>5	1.4	0.3–7.1	0.65	10.1	2.3–45	0.002

* Wald χ^2^.

Since the design of these studies was retrospective, a prospective study would be desirable. A post-hoc analysis of a prospective study, which had originally been performed to evaluate the role of dyslipidemia on the progression of renal failure in 73 patients with primary renal disease found that smoking status at entry was related to the decline in glomerular filtration rate after 3.2 years of follow-up [87]. In patients with chronic glomerulonephritis the loss of glomerular filtration rate was 5.3 mL/min/year in heavy smokers, but only 2.5 mL/min/year in non-smokers. Thus, smoking appears to double the rate of progression in patients with chronic glomerulonephritis. These data have to be interpreted with caution, because the level of statistical significance was not reached. This may be due to the short observation time and the small number of patients investigated.

The fact that we [61] and Stengel et al. [86] did not find an adverse effect of smoking on renal function in the small female subgroup is presumably related to the limited sensitivity of the studies; certainly the data do not permit to rule out an adverse effect of smoking on renal prognosis in women. Although speculation, it may be that females, at least prior to menopause, are less affected by the adverse renal effect of smoking. In favor of this hypothesis, one study including 246 patients with type 1 diabetes [88] found no relation between smoking and diabetic nephropathy in the female population (n = 106). In contrast, smoking males had a significantly increased risk to develop nephropathy. A further rational is that a recent biopsy study [89] found structural alterations of the kidney in male but not in female smokers (vide infra).

Only a few data are available concerning the effect of smoking on renal function in systemic diseases involving the kidney, namely lupus nephritis. A retrospective cohort study of 160 patients with a median follow-up of 6.4 years documented that smoking at the time of onset of lupus nephritis was an independent risk factor for more rapid progression to ESRF [90]. Life-table analysis was performed to calculate the median time interval to ESRF. It was 145 months in smokers and in excess of 273 months in non-smokers. This observation seems pertinent, because the effect of smoking was independent of hypertension and immunosuppressive treatment. These data have, however, not been confirmed in a recent prospective study including 70 consecutive patients with lupus nephritis [91]. In this latter study, the patients were compared to 70 age- and sex-matched controls with systemic lupus erythematosus without evidence of nephropathy. At the last visit of the 10 years of follow-up, 67% of lupus nephritis patients had normal plasma-creatinine, 24% had renal failure and 9% ESRF. Hyperlipidaemia and hypertension at study onset were the only factors associated with development of renal failure. Since no other studies have investigated this issue, the influence of smoking on prognosis of lupus nephritis remains unclear.

The hypothesis that heavy smoking might be a risk factor for the development and/or progression of pauci-immune ANCA-positive extracapillary glomerulonephritis has been forwarded [92], but solid data on this topic are missing. Due to the damaging effects of smoking on the vascular endothelium [93], it is conceivable that smoking-induced endothelial cell death and desquamation may predispose to the formation of antibodies against nuclear cell antigens extruded from endothelial cells or endothelial cell adherent polynuclear cells. Studies on this interesting and clinically relevant topic are needed.

As far as systemic diseases are concerned, cigarette smoking appears to be a risk factor for pulmonary complications in two clinical situations, which are of importance for the nephrologist. First, smoking increases the risk for fatal lung disease in hypocomplementemic urticarial vasculitis syndrome (HUVS) [94], a rare illness related to systemic lupus erythematosus. Second, the risk of pulmonary hemorrhage in anti-glomerular basement membrane (GBM)-disease, i.e. for the Goodpasture syndrome, is highly increased in smokers [95,96].

## Smoking and Atherosclerotic Renal Artery Stenosis/ischemic Nephropathy

The prevalence of atherosclerotic renal artery stenosis is increasing in the ageing population and ischemic nephropathy is a significant cause of ESRF in patients over 65 years of age [97].

The incidence of renal vascular stenosis increases as the extent of peripheral vascular disease increases [98]. Since the latter is common in smokers, it is not surprising that smokers have a higher risk of critical atherosclerotic renal artery stenosis [99]. Smoking is well known to promote atherogenesis. It is of interest that plasma total homocysteine concentration, a predictor of atherogenic risk, is strongly and dose-dependently related to cigarette smoking [100,101]. There is, however, no doubt that other pathogenic mechanisms also play a role.

Hadj-Abdelkader et al. [102] examined elderly hypertensive patients with renal failure by arteriography. A significantly higher proportion of patients with atherosclerotic renal artery stenosis, i.e. 80.5% were smokers compared to patients without atherosclerotic renal artery stenosis, i.e. 44%. A correlation was found with the number of cigarettes smoked and the exposure time. In a Spanish observational multicenter study 156 elderly patients with bilateral atherosclerotic renal artery stenosis and elevated serum-creatinine concentration were investigated. A high proportion, i.e. 70% were smokers [103]. As one would expect, the prevalence of smokers is increased both amongst patients with unilateral [104] and bilateral [105] atherosclerotic renal artery stenosis.

Although no reports are available about the rate of progression of renal failure in smokers versus non-smokers with renal artery stenosis/ischemic nephropathy, it is likely that smoking accelerates the course of renal failure. This assumption is based on the consideration that besides progressive narrowing of the renal artery, a combination of hypertensive and atheroembolic damage (i.e., cholesetrol embolism), is likely to contribute to progressive loss of renal function in patients with so-called ischemic nephropathy. Actually, smoking is a risk factor for cholesterol embolism [106-108].

In a group of 89 normotensive, non-diabetic elderly subjects with different degrees of peripheral atherosclerosis and no clinical signs of ischemic nephropathy, renovascular hypertension or other nephropathies, evaluation of renal function and plasma flow revealed that despite normal values for glomerular filtration rate, renal plasma flow declined progressively in parallel with the severity of peripheral atherosclerosis [109]. Stepwise multiple regression showed that the decrease in renal plasma flow was best explained by smoking and LDL-cholesterol [109]. Since there was a close association between the severity of extrarenal atherosclerosis and renal hypoperfusion, the authors concluded that the existence of initial ischemic nephropathy may be present in these subjects. These findings implicate that renal function should be assessed in patients with extrarenal atherosclerosis, particularly in those with classic cardiovascular risk factors.

## Adverse Effects of Smoking in Patients on Renal Replacement Therapy

Lower serum albumin concentrations predict increased mortality in hemodialysis patients. According to Wave-1 of the United States Renal Data System Dialysis Morbidity and Mortality special study, baseline serum albumin is significantly lower in active smokers as compared to non-smokers on hemodialysis [110]. It is of note that smoking is a highly significant risk factor of death during the first 90 days on hemodialysis [111]. Conversely, the prevalence of active smokers among long-term survivors on hemodialysis is low (11.8%) [112].

The analysis of 936 hemodialysis patients enrolled in the baseline phase of the Hemodialysis Study sponsored by the US National Institutes of Health revealed that diabetes and smoking are strongly associated with cardiovascular disease [113]. In a national random sample of new ESRD in the USA (n = 4,025), coronary artery disease was present in 38% of patients [114]. Of the total cohort, 17% had a history of myocardial infarction and 23% had angina. Several conventional risk factors, including advancing age, male gender, diabetes mellitus, and smoking, were significantly associated with coronary artery disease. Bypass surgery compared to angio-plasty yields better results in patients with ESRF. In this context, it is of importance to be aware of the fact that smoking results in a very poor outcome after bypass surgery. One retrospective study investigating 44 dialysis patients undergoing coronary artery bypass grafting from 1984 to 1997 reported a five-year survival of 0% for smokers and 83.6% ± 7.6% for non-smokers [115].

Smoking further adds to the increased mortality in patients with diabetes mellitus on hemodialysis, being an independent risk factor contributing particularly to cardiovascular death [47,49,116,117]. In one study, smoking and increasing age in patients with diabetes mellitus were the most important adverse features for outcome at the start of renal replacement therapy [118]. The relative risk for mortality in current cigarette smokers in this latter study was 2.28.

In dialysed patients with type 1 diabetes smoking has been shown to confer a relative risk for lethal myocardial infarction of 2.6 [119]. In a group of diabetic patients on hemodialysis (n = 22 smokers and n = 30 non-smokers), most of them having type 2 diabetes, the 5-year survival rate of the smoking patients was significantly decreased, i.e. 9% versus 37% in the non-smoking subjects. Cigarette smokers had higher fibrinogen and systolic blood pressure values. At the endpoint of the study, the incidence of myocardial infarction was significantly higher in the smoking patients (77% versus 13%). Cardiovascular events were the most frequent cause of death in both patient groups, but were more frequent in smokers (80% versus 63%) [120].

Smoking also confers a higher risk for atherosclerotic lesions outside the heart. In a study including 89 patients on hemodialysis and 30 on chronic ambulatory peritoneal dialysis, smoking correlated with the mean internal diameter of carotid arteries, the degree of carotid stenosis and the number of plaques in the carotid arteries [121]. An analysis of data from waves 1, 3 and 4 from the United States Renal Data System Dialysis Morbidity and Mortality Study documented that smoking is independently associated with peripheral vascular disease in hemodialysed patients (odds ratio up to 1.55) [122].

The risk of atrial fibrillation, a frequent arrythmia in hemodialysis patients, appears to be associated to coronary heart disease and may contribute to cardiovascular morbidity and mortality in ESRF [123]. Smoking per se does, however, not seem to be of importance for ventricular premature beats or complex ventricular arrhythmia in hemodialysis patients [124,125]. Of interest, smoking contributes, at least partly, to the decreased heart rate variability observed in patients with ESRF [126]. Furthermore, it is a risk factor for systolic dysfunction in dialysis patients [127]. As far as left ventricular function is concerned, a study including 217 non-diabetic dialysis and transplant patients revealed that besides high alkaline phosphatase (suggestive of hyperparathyroidism) and high serum creatinine levels (reflecting degree of uremia), smoking was the most significant and independent variable associated with low-output left ventricular failure [128].

According to a study from the U.S.A., which investigated a cohort of 1,572 patients (mean age 57.4 ± 15.0 years) who started hemodialysis in 1989, smoking is amongst the strongest predictors of the number of hospital days per year [129]. The major part of these patients were African Americans (63.7%) and 33% had diabetes mellitus as the primary cause of ESRF. Apart from cardiovascular and pulmonary complications, the increased number of hospital days per year in smokers on hemodialysis is probably also due to the the increased risk of early and late fistula failure [130].

Concerning patients on continuous peritoneal dialysis, only a few scattered informations about the adverse effects of smoking are available. Of importance, smoking increases the risk of permanent change to hemodialysis due to complications [131]. For non-diabetic patients, age, on treatment serum albumin, and current smoking were significant survival risk factors [132]. Whether smoking is a significant risk factor for the development of peripheral vascular disease in these patients, is controversial [133,134], although there is no plausible reason why it should not be.

In the context of management of the patient on renal replacement therapy, it is of importance that smoking is associated with incompliance in hemodialysis and peritoneal dialysis patients [135].

## Adverse Effects of Smoking in Patients with a Renal Transplant

Contraintuitively, it has been documented that smoking does not appear to increase the risk of microalbuminuria in patients with a renal transplant [136]. Most studies published to date indicated a lack of correlation of smoking with the development of progressive allograft dysfunction [137,138].

A recent cohort study of 645 adult renal allograft recipients performed from 1985 to 1995 evaluated the relationship between smoking and graft outcome [139]. Twenty-four percent of recipients (156/645) were smokers at the time of transplant evaluation. Of these, 90% continued to smoke after transplantation! Pretransplant smoking was significantly associated with reduced overall graft and death-censored graft survival. Patients who were smokers at the time of pretransplant evaluation had kidney graft survival of 84%, 65%, and 48% at 1, 5, and 10 years, respectively, compared with graft survival in non-smokers of 88%, 78%, and 62% (p = 0.007). Pretransplant smoking adversely affected death-censored graft survival in recipients of cadaveric (p = 0.02) and of living donor kidneys (p = 0.02). Reduced graft survival in pretransplant smokers could not be accounted for by differences in rejection episodes (64% versus 61%). In a multivariate analysis, pretransplant smoking was associated with a relative risk of 2.3 for graft loss. Among patients with a smoking history before transplantation, death-censored graft survival was significantly higher for those who quit smoking before transplant evaluation. Thus, this study documents that cigarette smoking before kidney transplantation contributes significantly to allograft loss, an effect that is not explained by increases in rejection episode or patient death. The finding that smoking cessation before renal transplantation has beneficial effects on graft survival is of major importance for the management of patients with ESRF who are considered for renal transplantation. In a retrospective analysis, current smoking has also been documented to be a risk factors for decreased graft survival in first-time kidney transplant recipients aged ≥ 60 years [140].

In an ongoing prospective study using the large Collaborative Transplant Study database, the issue is currently adressed by G Opelz (Heidelberg, Germany). A preliminary retrospective analysis of Opelz suggests that smoking by itself adversely affects late graft function, even if corrections are made for cardiovascular death with a functioning graft (personal communication). A similar analysis was performed by the group of LC Paul and yielded the same results [141]. It is likely that the final results of these investigations will confirm the data of Sung et al. [139].

The effect of smoking on renal allograft function may depend on the renal disease that has led to ESRF. In patients who had reached ESRF as a result of lupus nephritis, the risk of renal transplant loss was substantially increased in smokers [142]. In this study, smoking demonstrated both the strongest association and the highest relative risk for allograft loss (relative risk 2.5, p < 0.0001) as compared to the other factors which confered an increased risk for allograft loss, i.e., delayed graft function, acute rejection episodes, and total HLA mismatches. Lupus nephritis accounts only for a small proportion of patients requiring renal transplants, but the above results are of major clinical importance and point to the possibility that the alterations of the immune response reported in smokers [5] may be particularly detrimental in patients with immunoregulatory abnormalities such as systemic lupus erythematosus.

It is of note that an investigation of kidney donor lifestyle factors, including smoking, drinking, drug use, and sexual history, found no significant negative impact on renal allograft survival [143].

The evidence that the risk of death with a functioning graft is increased in patients with a history of cigarette smoking is beyond any doubt. The magnitude of the negative impact of smoking in renal transplant recipients is quantitatively similar to that of diabetes mellitus [144]. A retrospective analysis [145] found a higher rate of cardiovascular death with a functioning graft. This has also been documented in first-time kidney transplant recipients aged ≥ 60 years [140]. Compared to never-or ex-smokers, the diabetic renal transplant recipient had a significantly increased risk of early death if he smoked during the predialysis phase or after having received his graft [49].

The increase in cardiovascular death is due to the well known atherogenic effects of smoking [146,147]. Smoking appears to increase the risk for low-output left ventricular failure in patients with a renal transplant [128]. As one would expect, cigarette smoking at the time of transplantation is an independent risk factor for cerebrovascular and peripheral vascular disease in transplanted patients [148]. Smoking post-transplantation is a risk factor for carotid plaques [149] and peripheral vascular occlusive disease [150].

Diabetic transplant recipients are at a high risk for foot pathology leading to amputation. Smoking has a profoundly negative effect on the amputation rate as has an amputation prior to transplantation [151].

Another clinically relevant aspect is the higher incidence of cancer in smokers. In one study, cigarette smoking was associated with an increased risk for cancer, with each 10 pack-years smoked at transplant increasing the risk by 1.12 (1.02–1.21; p = 0.016) [152]. It has also been reported that smoking increases the risk for squamous cell carcinoma of the skin [153]. Furthermore, exposure to the sun and smoking are risk factors for dysplastic and malignant lip lesions in renal transplant recipients [154].

Finally, smoking increases the risk for osteoporosis in corticosteroid-treated transplant patients (and patients with chronic glomerulonephritis) [155] and favours the development of post-transplant erythrocytosis [156].

## Potential Mechanisms of Smoking-induced Renal Damage

Several potential mechanisms of smoking-induced renal damage have been discussed in detail elsewhere [7] and are summarised in Table 4. These include acute effects, particularly sympathetic activation (influencing blood pressure and renal hemodynamics), and chronic effects, particularly endothelial cell dysfunction (diminished nitric oxide availability, diminished endothelial cell-dependent vasodilation, and intimal cell hyperplasia). The precise nature of the nephrotoxic effect of smoking is, however, far from being understood. It will be particularly difficult, probably impossible, to delineate which substance(s) in tobacco smoke is (are) responsible for the adverse renal effects of smoking.

**Table 4:** Potential pathomechanisms of smoking-induced renal injury

• Increased sympathetic nerve activity
• Increase of blood pressure and heart rate
• Decreased fall of night-time blood pressure
• Increase of renal vascular resistance leading to a decrease in glomerular filtration rate and renal plasma flow
• Increase of intraglomerular capillary pressure
• Aggravation of hyperfiltration in patients with diabetic nephropathy
• Atherosclerosis of renal arteries and myointimal hyperplasia of the intrarenal arteries and arterioles
• Endothelin-1- and/or angiotensin II-mediated proliferation and matrix accumulation of vascular smooth muscle cells, endothelial cells and mesangial cells
• Tubulotoxic effects with alteration of tubular function
• Toxic effects on endothelial cells
• Oxidative stress
• Increased clotting of platelets
• Impaired lipoprotein and glycosaminoglycan metabolism
• Modulation of the immune response
• Vasopressin-mediated antidiuresis
• Insulin resistance

Some pathomechanisms of smoking-induced renal damage, which seem of particular importance, are briefly discussed in the following.

### Increase in sympathetic activity and blood pressure

Since the first decade of the 20th century it has been known that smoking induces a transient increase of BP and heart rate [157]. The increase in BP and heart rate seems to be related to nicotine per se, since no such changes occur when nicotine-free cigarettes are smoked [158]. Today we know that these acute hemodynamic effects are mediated mainly via sympathetic activation and vasopressin release. In addition, in patients with primary hypertension an increase in cortisol, adrenocorticotrophic hormone (ACTH) and plasma aldosterone concentration has been noted during smoking [7].

Grassi et al. [159] demonstrated that nicotine increases sympathetic activity via direct stimulation of postganglionic sympathetic nerve endings: smoking a single cigarette markedly increased plasma concentrations of norepinephrine and epinephrine in healthy volunteers, whereas postganglionic muscle sympathetic nerve traffic decreased significantly. Thus, nicotine directly stimulates catecholamine release from peripheral sympathetic nerve endings and the adrenal medulla [160]. Increased sympathetic activity may also accelerate progression of renal failure [161]. Thus, it is plausible to assume that a further increase of catecholamine release from peripheral sympathetic nerve endings induced by smoking in subjects with renal disease may damage the kidney via two mechanisms, i.e., indirectly through blood pressure elevation, but also as a direct result of activation of the sympathetic system.

In view of the importance of blood pressure on the evolution of renal disease, the effects of smoking on blood pressure are of considerable interest. Ambulatory BP measurements documented that smoking in parallel with the stimulation of the sympathetic system causes a significant, but transient increase (lasting ~30 minutes) of blood pressure in healthy [162] and hypertensive subjects [163,164]. This was also found in patients with type 1 [165,166] and type 2 diabetes [167] or primary renal disease [168].

Smoking also seems to alter the diurnal rhythm of blood pressure. Hansen et al. [169] reported that the night/day ratio of systolic and diastolic blood pressure in healthy smokers was lower than in non-smokers. In one study a derangement of the diurnal rhythm of blood pressure was not found in patients with type 1 diabetes who smoked. In contrast, a preliminary communication [170] documented decreased ratios of daytime to nighttime blood pressure in both smoking healthy volunteers and in subjects with type 1 diabetes. It is probable that the presence or absence of autonomic neuropathy in patients with diabetes mellitus is responsible for these contrasting results. For instance, in patients with type 1 diabetes smoking increases systolic blood pressure only in subjects without autonomic neuropathy [166].

### Alteration of intrarenal hemodynamics

Ritz et al. [168] performed a study in healthy volunteers with a history of limited cigarette consumption (<10 cigarettes/d) to investigate the effects of smoking on the healthy kidney: smoking (as compared to sham-smoking) caused a significant decrease in glomerular filtration rate (GFR), filtration fraction (FF) and renal plasma flow (RPF) as measured by radioisotope infusion clearance. Renovascular resistance increased significantly. The FF is a surrogate marker of glomerular capillary pressure. This parameter decreased in healthy volunteers. At first sight this observation may appear paradoxical, since reduced intraglomerular pressure should provide nephroprotection, but the renal response is apparently different in subjects with and without renal disease. The findings of Ritz et al. concerning GFR and RPF were confirmed by Halimi et al. [171] in non-smokers who chewed a nicotine chewing gum. Interestingly, in this letter study renal vasoconstriction did not occur in smokers. The authors concluded that smokers continue to exhibit the systemic response to nicotine, i.e., an increase in blood pressure and heart rate, but are tolerant to the renal effects of nicotine. The latter was attributed to a compensatory increase of the synthesis of cyclic guanosine monophosphate (cGMP) in the kidney of smokers as indicated by increased urinary excretion. In a cross-sectional study in 30 subjects with no known vascular risk factors other than chronic cigarette smoking and 24 age- and sex-matched non-smokers without any vascular risk factors, Gambaro et al. [172] reported that smokers had a significant reduction of RPF, but normal GFR. Thus, there is no doubt that acute and chronic smoking induces renal functional alterations, which differ slightly according to the experimental setting.

Ritz et al. [168] also compared the renal hemodynamic effects of smoking in volunteers with those in patients with IgA-glomerulonephritis. Whilst the increase in mean arterial pressure and heart rate was similar in patients with IgA-glomerulonephritis and volunteers, a significant decrease in GFR and FF was not demonstrable in patients with IgA-glomerulonephritis, in contrast to what was seen in volunteers. During smoking, a significant increase in the urinary albumin/creatinine ratio was noted, further pointing to an adverse effect of smoking on glomerular capillary pressure.

**Figure 1:**
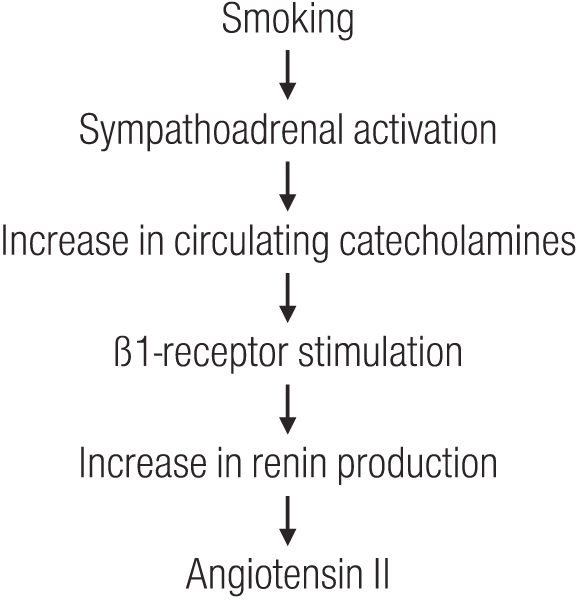
**Hypothetical sequence of smoking-induced activation of the renin-angiotensin system via activation of the sympathetic nervous system as one major pathomechanism of smoking-induced renal damage**.

Taken together these findings are consistent with the hypothesis that in patients with glomerular disease, in whom the preglomerular vasculature is presumably vasodilated, smoking-induced vasoconstriction is unable to overcome vasodilation. As a result one would expect that the increase in systemic pressure is transmitted partially to the glomerular microcirculation, causing acute glomerular hypertension. As reported by Benck et al. [173] pretreatment with the a-adrenergic blocker prazosin (compared to placebo) failed to affect the change in renal hemodynamics, whilst pretreatment with the β-blocker atenolol obliterated the renal hemodynamic response. This finding together with the observation of an abrogation of the adverse renal effect of smoking by ACE inhibitor treatment in patients with primary renal disease [61] is consistent with the hypothesis depicted in Figure 1.

### Oxidative stress

Oxidative stress is probably another major player in the genesis of smoking-induced vascular renal injury. Extrusion of glutathione from endothelial cells and activation of the hexose monophosphate shunt, which is necessary to maintain glutathione in the reduced state, point to the presence of oxidative stress, which may be imposed by the free radicals that are present in tobacco smoke [174]. The concentrations of antioxidant enzymes such as glutathione peroxidase, catalase and superoxide dismutase decrease and markers of oxidative stress increase as renal insufficiency advances [175]. The patient with renal failure may thus be particularly susceptible to additional oxidative stress induced by smoking.

### Smoking-induced nitric oxide depletion

The inhibitory effect of smoking on nitric oxide generation may play a critical role in increasing renal vasculature tone. In addition, intrarenal arterial dilation in response to nitroglycerine is significantly impaired in type 2 diabetic patients who smoke [176]. Nitric oxide depletion in smokers may also promote smooth vascular cell and mesangial cell proliferation [177,178]. A genetic approach to explain the different susceptibility of individuals to smoking-induced organ damage has been proposed by Wang et al. [179]: the risk of atherogenesis appears to be excessively high in patients who are homozygous for the endothelial nitric oxide synthase 4a (ecNOS4a) gene. This genotype predisposes to endothelial dysfunction and is associated with an increased coronary risk in smokers. Whether a similar genetic susceptibility determines an increased renal risk in smokers is an issue that deserves further investigation.

## Pathohistological Features of Smoking-induced Renal Damage

An increase in thickness of walls of arterioles of organs not in direct contact with cigarette smoke, mainly due to fibroelastic intimal proliferation and hyaline thickening in the intima, has been observed in various organs of individuals without renal disease [180,181], including the kidney [182-184].

In a renal biopsy study, the histological findings of 107 patients (aged 48 ± 12 years) with chronic renal failure were assessed by investigating the effect of smoking on glomerulosclerosis and vascular damage [89]. Most of these patients were suffering from glomerular disease with marked proteinuria, only a minority had been treated with an ACE inhibitor at the time of biopsy and blood pressure was not well controlled (152/91 mmHg). Smoking was not associated with glomerulosclerosis. As compared to non-smokers, ever-smokers exhibited more severe myointimal hyperplasia. This finding was particularly evident in patients over 50 years of age. In younger patients, a trend toward arteriolar changes was evident for smoking, but it did not reach statistical significance. In females, no correlation was observed. This may due to the fact that women were less likely to be smokers and smoked less than half as many pack-years than did men.

The above study is important, because it documents that ever-smoking increases myointimal hyperplasia in elderly male patients with renal disease. Since hypertension per se seems not to be related to myointimal hyperplasia of intrarenal arterioles [185], the effect of smoking has to be considered as particularly important. The negative finding concerning glomerulosclerosis does not exclude a negative effect of smoking on glomerular structure. Using a more precise method for quantification of renal damage, our group found more severe glomerulosclerosis and tubulointerstitial fibrosis in a rat model of focal-segmental glomerulosclerosis, i.e. the subtotally nephrectomised rat [186]. Whether this is true for humans with non-inflammatory renal disease as well remains to be determined. An increase in glomerular basement width in patients with type 2 diabetes who smoke has been reported in a preliminary study [187]. A rare form of glomerulosclerosis, i.e. idiopathic nodular glomerulosclerosis, has been reported to be observed almost exclusively in smokers. In this latter renal biopsy study including 5,073 patients, 23 cases of idiopathic nodular glomerulosclerosis were diagnosed. Ninety-one percent of these cases were heavy smokers (52.9+6.9 pack years) and 96% had hypertension. Seventeen of the patients were followed-up and 6 reached ESRF after a mean of 8.7 months. Importantly, cessation of smoking significantly improved renal prognosis.

## Reversibility of Smoking-induced Renal Damage

The above observation in patients with idiopathic nodular glomerulosclerosis or a renal transplant indicate that cessation of smoking may substantially reduce the rate of progression of renal failure. The question arises whether this is also true in patients with common renal diseases.

One study in patients with type 1 diabetes and nephropathy provided convincing evidence in this respect [35]: in patients with adequate control of blood pressure, cessation of smoking significantly decreased urinary albumin excretion, although glycemia was not perfectly controlled. In another study, progression was found in 53% of current smokers, 33% of ex-smokers and 11% of non-smokers [53].

It is plausible to assume that this may also be true in non-diabetic renal disease. Pinto-Sietsma et al. [9] found that the risk of microalbuminuria in non-diabetic subjects is only minor in ex-smokers, but not in current smokers. There is some evidence, however, that smoking-induced decrease in renal plasma flow is not completely reversible after smoking cessation [172].

The present data do not allow to draw a definite conclusion about the magnitude of the renal benefit derived from smoking cessation. There is, however, good evidence indicating that smoking cessation is one of the single most effective measures to retard progression of renal failure.

## Conclusion

Smoking is one of the most important remediable renal risk factors. It has a negative impact on renal function even in subjects without apparent renal disease, but the adverse renal effects of smoking are particularly eminent in patients with a diseased kidney. Importantly, the increase in the rate of progression of renal failure attributable to smoking seems to be independent of the underlying renal disease.

Nephrologists have to be aware of two major growing medical problems. First, the increase in the number of patients requiring renal replacement therapy, which is partly due to cigarette smoking. Second, the dramatic increase of deaths related to cigarette smoking. The World Health Organization estimated that world-wide tobacco abuse accounted for 3 million deaths in 1996 and even 10 million deaths are expected for the year 2020 [189].

Besides improvement of renal prognosis, cessation of smoking undoubtedly improves cardiovascular prognosis in the renal patient [6]. Thus, Even if ESRF is reached, smoking should be discontinued. Smoking is the strongest predictor of mortality in type 2 diabetes [6], the fastest growing population reaching ESRF. According to lifetable analyses, smoking cessation prolongs the life of a 45-year-old smoking, hypertensive, diabetic male by 4–5 years; the treatment of hypertension is estimated to prolong the life of the same individual by only 1 year [190]. Of note, even among people who have a history of heavy smoking, the risk of coronary events can be halved by stopping the habit. This benefit from cessation of smoking is seen regardless of how long or how much a person has previously smoked [191]. It has been stated that "persuading hypertensive patients not to smoke is the single most effective measure we can take to reduce their risk" [192]. According to estimates the risk of myocardial infarction can be reduced by 50–70% as a consequence of cessation of smoking. In contrast, the treatment of hypertension results in a reduction of risk of myocardial infarction of "only" 2–3% for each 1 mmHg decline in diastolic BP [193].

Tobacco control programs with the ultimate goal to reduce tobacco use by young individuals are effective [194]. Besides such important preventive measures, major efforts have to be undertaken to help patients to quit smoking. These include the most effective pharmaceutical smoking cessation approaches known to date, i.e. therapy with sustained-release bupropion and nicotine replacement therapy [195,196].

Management of the renal patient requires information about (i) the magnitude of the renal and cardiovascular risk related to smoking including the benefits from smoking cessation and (ii) application of the above modern therapeutic modalities to increase the success rate in patients willing to stop smoking. To the best of my knowledge, there is no information about the exact pharmacokinetics of sustained-release bupropion in patients with impaired renal function. Apparently, bupropion does not accumulate in renal failure. In contrast, nicotine accumulates in renal failure [197], a fact which has to be acknowledged when treating patients with nicotine replacement therapy. Accumulation of nicotine may limit the success rate of smoking discontinuation in patients with renal failure, because the patient is used to a higher level of "intoxication". Actually, diabetic patients with nephropathy have smoked more and still smoke more than patients without nephropathy [48]. On the other hand, it is plausible to assume that more renal patients than individuals in the general population will stop smoking, because they are afraid of the prospect to progress to ESRF. This assumption of greater compliance in severely ill patients is based on the observation that in subjects receiving intervention to stop smoking one year smoking cessation rate is approximately 35% in healthy subjects [195] but approximately 70% in patients after a myocardial infarction [198]. It has to be pointed out that to date, no data are available about the success of a modern smoking cessation strategy in renal patients.

## Note Added in Proof

As discussed on page 142, males may be more susceptible to smoking-induced renal damage than females. Recent data strengthen this hypothesis [199]: A population-based, cross-sectional study [200] of 11,247 Australian adults revealed that smoking was independently associated with renal impairment in men with an odds ratio of 3.59, but not in women. Smoking was significantly associated with proteinuria in subjects with high-normal systolic blood pressure, with odds ratios ranging from 3.64 at 131.5 mm Hg to 5.76 at 139.5 mm Hg, and in subjects with high-normal 2-hour glucose levels, with odds ratios ranging from 1.76 at 7.0 mmol/L to 10.84 at 7.7 mmol/L. Lifetime exposure, but not current level of smoking, correlated with lower estimated glomerular filtration rate and greater urine protein-creatinine ratio. Tozawa et al. [201] investigated a large Japanese population (n = 5,403) and found that smoking conferred a relative risk of 1.28 for developing proteinuria in men, but it did not increase the risk in women.

## Competing interests

The authors declare that they have no competing interests.
